# Inner ear salivary gland choristoma extending to the middle ear with congenital profound hearing loss and facial palsy: a case report

**DOI:** 10.1186/s40463-021-00511-3

**Published:** 2021-04-15

**Authors:** Yoshiharu Yamanobe, Naoki Oishi, Takanori Nishiyama, Makoto Hosoya, Kaoru Ogawa

**Affiliations:** Department of Otolaryngology-Head and Neck Surgery, Keio University School of Medicine, 35 Shinanomachi, Shinjuku-ku, Tokyo, 160-8582 Japan

**Keywords:** Salivary gland choristoma, Inner ear tumor, Congenital hearing loss, Pediatric ear tumors, Pediatric facial palsy, Disequilibrium

## Abstract

**Background:**

Salivary gland choristoma (SGCh) is a rare benign tumor reported in several unusual sites, such as the gastrointestinal tract, the optic nerve, and the internal auditory canal, but never reported in the inner ear.

**Case presentation:**

An 8-year-old girl with a history of left profound congenital hearing loss presented to us with ipsilateral progressive severe facial nerve palsy (House-Brackmann Grade VI). The left tympanic membrane was swollen with a pulsatile tumor. Radiological investigations revealed a multilocular tumor in the inner ear extending into the middle ear and internal auditory canal (IAC). We performed a partial resection of the tumor by transmastoid approach to preserve the anatomical structure of the facial nerve. The tumor was pathologically diagnosed as SGCh. Two years after surgery, her facial function recovered to House-Brackmann Grade II and the residual tumor did not show regrowth on MRI.

**Conclusions:**

Although the natural course of this rare tumor is unknown, a partial resection is an acceptable treatment procedure when functional recovery of the facial nerve is anticipated.

**Graphical abstract:**

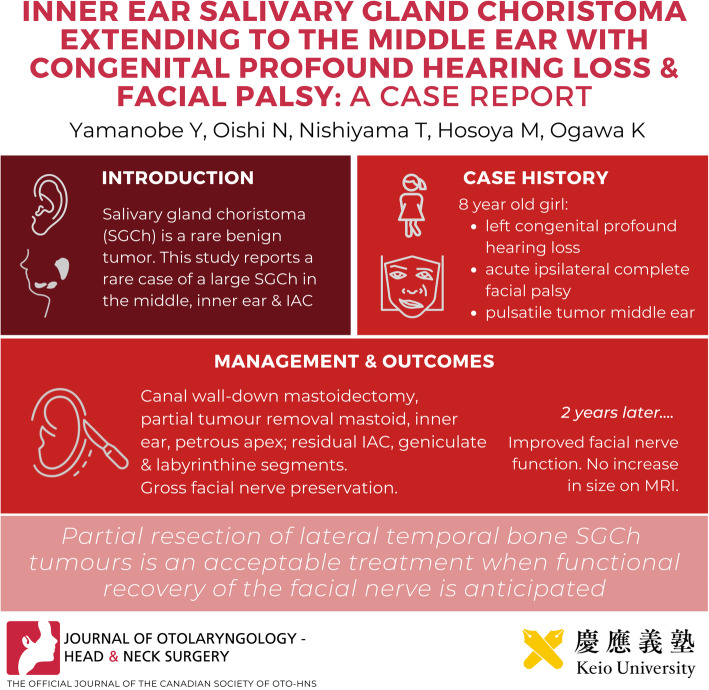

## Background

Salivary gland choristoma (SGCh) is a rare benign tumor characterized by a histopathological finding of normal salivary tissue, mucous and mixed predilection, and occasionally, fibrosis [1]. SGCh is distinguished from ectopic salivary glands which are present in abnormal locations without fibrous capsules [2]. The primary sites of SGCh have been reported in several organs, such as the gastrointestinal tract, the optic nerve, and the internal auditory canal [3, 4]. SGCh in the middle ear can sometimes be accompanied by an aberrant facial nerve and middle ear malformations [5–7]. Here, we report an extremely rare case of a large inner ear SGCh extending into the middle ear and the internal auditory canal (IAC), with congenital unilateral profound hearing loss and ipsilateral progressive facial nerve palsy.

## Case presentation

An 8-year-old girl with a history of congenital profound hearing loss in the left ear presented to a local ENT hospital with an acute onset of ipsilateral facial palsy. She was treated with oral prednisolone; however, the facial palsy progressed to complete paralysis. Otoscopy revealed a pulsatile mass in the left middle ear. A detailed medical history revealed that she had been able to perform tandem walking at the age of four, but became unable to do so at the age of six. She had never been examined by MRI to find the cause of congenital hearing loss.

**Fig. 1:**
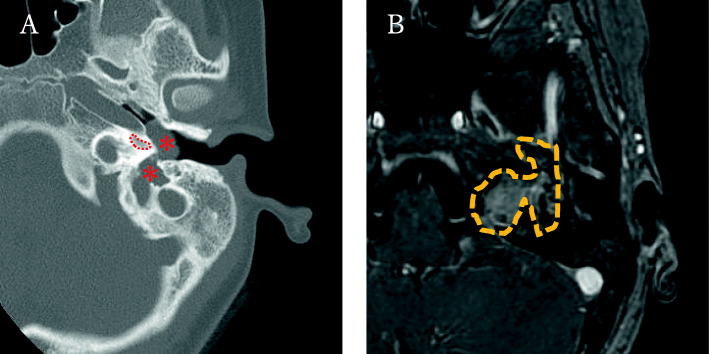
**a** Preoperative CT and MRI finding. CT shows the tumor was found as a continuous soft shadow from the inner ear to the middle ear by tumor (*). (Dotted area: cochlea). **b** MRI shows the tumor (dotted area) extends from the inner ear to the middle ear as well as the internal auditory canal, presenting as low signal on contrasted T1

**Fig. 2:**
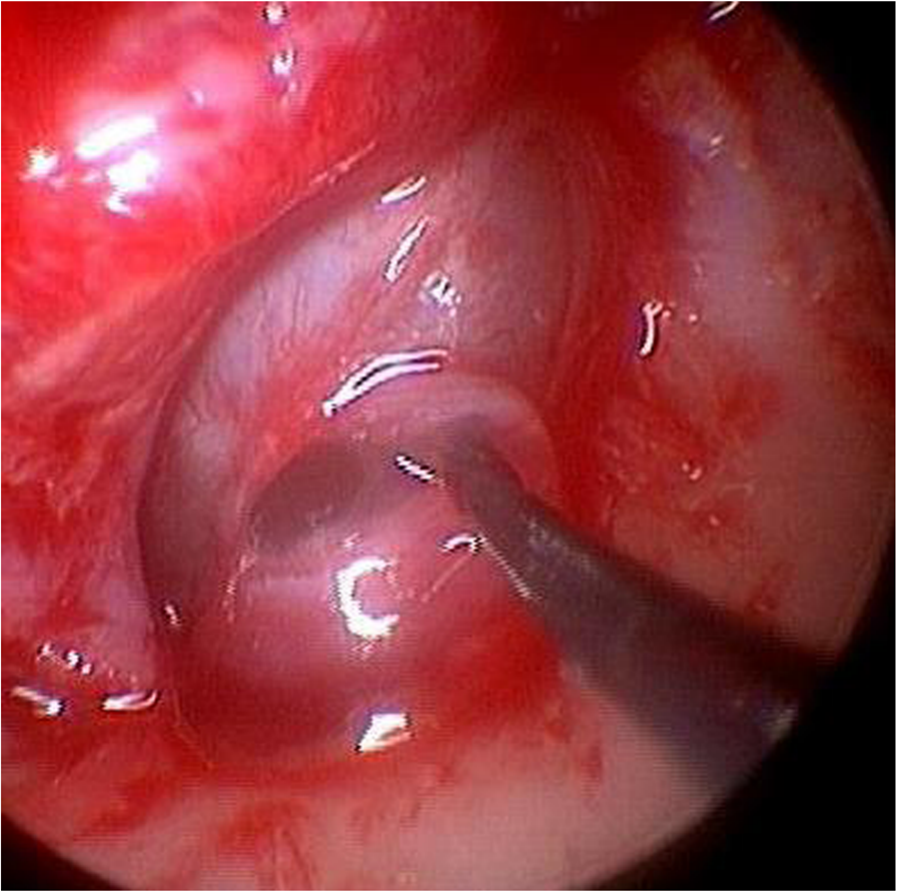
Intraoperative findings of endoscopic transcanal biopsy. The tumor was found right after the elevation of the tympanic membrane (*). Mucinous fluid exuded from the inside of the tumor

On physical examination, she had a left complete facial paralysis (House-Brackmann grade VI). Electroneurography showed no visible reaction on the affected side (0%). Her left tympanic membrane was swollen, and a pulsatile tumor was visible in the middle ear, which was first found since the diagnosis of congenital hearing loss. Pure tone audiometry showed profound hearing loss in the left ear and less than 20 dB in the right ear. Auditory brainstem response done at the age of five had revealed 100 dB hearing threshold in the left ear, and auditory steady-state response had showed hearing thresholds of the left ear as 80 dB at 1000 Hz, 85 dB at 2000 Hz, and 90 dB at 4000 Hz. Computed tomography (CT) examination revealed a multilocular tumor in the inner ear extending into the middle ear (Fig. 1a), and osteosclerosis around the edges of the tumor. In the middle ear, the tumor pressed on the ossicles, and the facial nerve canal could not be identified. On MRI, the tumor presented mixed signals of high and low intensities on T2-weighted images and was sparsely enhanced with gadolinium on T1-weighted images (Fig. 1b). We performed an endoscopic transcanal biopsy of the tumor. It was soft, with a thick fibrous capsule and filled with mucinous fluid (Fig. 2). The pathological findings revealed only blood components in the tissue fluid, and the biopsy specimen revealed collagen fibers, smooth muscle, and small blood vessels.

**Fig. 3:**
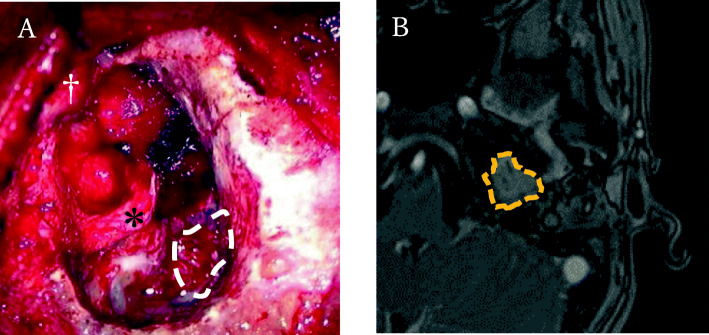
**a** At the end of the surgical removal. The tumor was spared in the internal auditory canal and the deep layer of the epitympanum (white dotted area: residual tumor in the internal auditory canal). *: facial nerve; †: temporomandibular joint). **b** MRI at 2 years postoperatively shows a residual lesion of about 2 cm in the deep part of the left petrous bone near the internal auditory canal up to the epitympanum (dotted area), which did not show regrowth compared to the MRI images at 2 months after the surgery

Subsequently, we decided to perform a transmastoid tumor removal with facial nerve monitoring using nerve integrity monitoring. A retroauricular incision was made, the external auditory canal (EAC) and the tympanic membrane were removed, and a blind sac closure of the EAC and canal wall down mastoidectomy were performed. A hemorrhagic mass was visualized filling the middle ear toward the epitympanum. We removed most of the tumor in the mastoid cavity, the inner ear, and the petrous apex, and the facial nerve was completely dissected and delineated from the horizontal to the vertical portion. However, we decided to spare the tumor in the deep part near the epitympanum and the internal auditory canal because facial nerve monitoring signals were completely lost, and identification of the geniculate ganglion and the labyrinthine portion of the facial nerve was impossible as the tumor had engulfed the nerve at these regions. Anatomical preservation of the facial nerve was considered significant to retain any chances for recovery of the facial palsy after surgery (Fig. 3a). No intraoperative or postoperative complication such as spinal fluid leakage was observed. Pathological diagnosis showed a malformed salivary gland structures consisting of smooth muscle tissue proliferation on immunostaining, which led to the diagnosis of SGCh. One year after surgery, the palsy recovered to House-Brackmann Grade II. Two years after surgery, MRI showed no increase in size of the residual tumor (Fig. 3b).

## Discussion

We have reported an unusual case of pediatric SGCh with extensive involvement of the inner and middle ear. The definitive diagnosis of this case was different from our preoperative diagnosis. Our differential diagnoses for this pediatric inner ear tumors included facial schwannoma, endolymphatic sac tumors, vestibular schwannoma, and hemangiomas. A preoperative diagnosis of SGCh is difficult considering the very low frequency of these tumors [8]. Though the etiology of SGCh remains controversial, many reports suggest that they are caused by abnormal differentiation of the first and second branchial clefts in the fetal period. The development of salivary glands begins at 4–12 weeks when the thickening of the oral epithelium of the first branchial cleft results in an epithelial bud, around which the ectodermal mesenchyme cells assemble. The epithelial buds repeatedly divide and eventually form the capsules and septum of the salivary glands. However, the over proliferating capsular epithelium can stray into the inner ear tissue and get isolated. It has also been suggested that SGCh in the middle ear causes middle ear malformations and aberrant deviations of the facial nerve canal [5]. The primary site of SGCh varies, the usual sites being the eyes, optic nerve, and the internal auditory canal. However, the number of cases reported in these sites are very low [7–9].

We had reasons to consider that the inner ear was the primary site in this case. First, the patient had a congenital profound hearing loss. Second, no previous abnormalities in the tympanic membrane had been detected. Third, imaging studies suggested that the tumor was mainly located in the inner ear.

The treatment of SGCh involves removal of the tumor. However, SGCh is rare and there are no specific guidelines for their treatment. The pathological features of SGCh are believed to be benign and do not transform to malignancy [10], but there have been a few reports of malignant middle ear SGCh [11]. We concluded that this case required surgical resection since the biopsy did not yield the pathological diagnosis and the tumor seemed to be growing with progressive clinical symptoms such as balance dysfunction and facial paralysis. We removed most of the tumor, however, we could not remove all the tumors in the deep part near the epitympanum and the IAC because identification of the geniculate ganglion and the labyrinthine portion of the facial nerve was impossible.

In this case, the transmastoid approach and removal of the external auditory canal with blind sac closure were selected mainly to watch the tumor in wide range. The blind sac closure of the meatus had some disadvantage such as making it impossible to observe residual tumor in the mastoid cavity through the meatus postoperatively. However, removing the meatus gave a better view of the tumor especially in the middle ear and the petrous apex. Regarding the transmastoid approach, a previous adult case of a small SGCh in the IAC was removed using a retro-sigmoidal approach with preservation of facial nerve function [3]. However, the retro-sigmoidal approach was not a good choice in our case with a tumor extending to the middle ear and no preoperative hearing function. We believe that our surgical strategy in this case was appropriate because good tumor control and recovery of facial nerve function from House Brackman Grade VI to Grade II were achieved without any complications such as cerebrospinal fluid leakage. The residual tumor needs to be carefully monitored in future using MRI, and total removal of the tumor through transotic approach may be necessary if there is an increase in the size of the residual tumor.

## Conclusion

Although the natural course of this rare tumor is unknown, a partial resection is an acceptable treatment procedure when functional recovery of the facial nerve is anticipated, especially in the pediatric cases.

## Data Availability

Not applicable

## Abbreviations

SGCh: Salivary gland choristoma
IAC: Internal auditory canal
EAC: External auditory canal
